# Complementary UV-Absorption of Mycosporine-like Amino Acids and Scytonemin is Responsible for the UV-Insensitivity of Photosynthesis in *Nostoc flagelliforme*

**DOI:** 10.3390/md8010106

**Published:** 2010-01-20

**Authors:** Lorenzo Ferroni, Manfred Klisch, Simonetta Pancaldi, Donat-Peter Häder

**Affiliations:** 1 Laboratory of Plant Cytophysiology, Department of Biology and Evolution, University of Ferrara, Corso Ercole I d’Este, 32, 44100 Ferrara, Italy; E-Mails: lorenzo.ferroni@unife.it (L.F.); simonetta.pancaldi@unife.it (S.P.); 2 Department for Biology, Friedrich-Alexander University Erlangen-Nuremberg, Staudtstraße 5, 91058 Erlangen, Germany; E-Mail: mklisch@biologie.uni-erlangen.de (M.K.)

**Keywords:** mycosporine-like amino acids, Nostoc flagelliforme, scytonemin, photosynthesis, UV-radiation

## Abstract

Mycosporine-like amino acids (MAAs) and scytonemin are UV-screening compounds that have presumably appeared early in the history of life and are widespread in cyanobacteria. Natural colonies of the UV-insensitive *Nostoc flagelliforme* were found to be especially rich in MAAs (32.1 mg g DW^−1^), concentrated in the glycan sheath together with scytonemin. MAAs are present in the form of oligosaccharide-linked molecules. Photosystem II activity, measured using PAM fluorescence and oxygen evolution, was used as a most sensitive physiological parameter to analyse the effectiveness of UV-protection. Laboratory experiments were performed under controlled conditions with a simulated solar radiation specifically deprived of UV-wavebands with cut-off filters (295, 305, 320, 345 and 395 nm). The UV-insensitivity of *N. flagelliforme* was found to cover the whole UV-A (315–400 nm) and UV-B (280–320 nm) range and is almost certainly due to the complementary UV-absorption of MAAs and scytonemin. The experimental approach used is proposed to be suitable for the comparison of the UV-protection ability in organisms that differ in their complement of UV-sunscreen compounds. Furthermore, this study performed with a genuinely terrestrial organism points to the relevance of marine photoprotective compounds for life on Earth, especially for the colonization of terrestrial environments.

## 1. Introduction

The synthesis of organic compounds that show strong absorption in the UV-range is widely distributed among the life on Earth [1]. Well-known examples of such compounds are melanin in humans and numerous other organisms including animals, fungi and bacteria, phenylpropanoids in plants [2] and mycosporine-like amino acids (MAAs) in eukaryotic algae and cyanobacteria [2–4]. MAAs are water-soluble compounds of low molecular weight [3], composed of either an aminocyclohexenone or an aminocyclohexenimine ring, bearing nitrogen or aminoalcohol substituents [5]. These compounds are found in marine, freshwater and, to a smaller degree, in terrestrial species [6]. Typically MAAs are intracellular compounds; however, in some cyanobacteria extracellular oligosaccharide-linked MAAs (OS-MAAs) may also occur. In these compounds, the MAA chromophore is linked to oligosaccharide side chains leading to molecules that strongly interact with extracellular polysaccharides and proteins [7,8]. Among natural products, MAAs have been considered interesting candidates as sunscreen compounds because of their outstanding UV-absorption, coupled with a high photostability [6]. Indeed, algal extracts containing MAAs are commercialized by several companies as base materials in cosmetic products for skin protection against UV radiation, being considered a safe alternative to synthetic UV-sunscreens (e.g., Helioguard 365^™^, Helionori^™^). In the extracellular sheath of certain cyanobacteria the lipid-soluble yellow-brown pigment scytonemin is another important UV-screening compound [9]. This substance is also an inhibitor of polo-like kinase 1 activity and of various other cell cycle-regulatory kinases and has been proposed to be useful as a template for the development of more potent and selective kinase inhibitors that can be used for the treatment of hyperproliferative disorders [10].

The accumulation of UV-screening compounds clearly represents an adaptive benefit against the harmful effects of UV radiation, which, especially the highly energetic UV-B waveband (280–315 nm), affects organisms in several ways. Among the adverse effects are DNA-damage by the formation of thymine dimers [11], DNA strand breaks and lipid peroxidation [12], and impairment of motility and orientation [13]. In photosynthetic organisms, a well-known target of UV-radiation is photosystem II (PSII), the fundamental enzymatic machinery responsible for the initiation of the photosynthetic electron transport chain [14]. For organisms that undergo periods of physiological inactivity (e.g., desiccation), passive defence mechanisms such as the screening of UV-radiation are of utmost importance, because the active repair mechanisms that depend on metabolic activity are ineffective under these conditions [15].

An example of outstanding UV-insensitivity of photosynthesis has been described in *Nostoc flagelliforme* [16]. This is a terrestrial cyanobacterium that has been known by the Chinese for centuries for its edible and medicinal values. It thrives in arid or semi-arid environments characterized by low annual precipitation, high evaporation and intense solar radiation [17], where it undergoes frequent cycles of desiccation and rehydration [18]. Exposure to strong solar radiation and frequent changes in the water status find interesting parallels in the littoral zones of marine habitats. The desiccated form of *N. flagelliforme*, which has a hair-like appearance, upon rewetting undergoes a quite rapid resumption of the photosynthetic activity [19]. Many studies have been carried out on the ecophysiology of *N. flagelliforme* in recent years [16–22]. Gao and Ye have recently reported the photosynthetic insensitivity of this organism to solar UV-radiation during rehydration and desiccation [16].

In the present study, we investigated the UV-absorbing compounds accumulated in natural colonies (trichomes) of *N. flagelliforme*. The presence and concentration of the compounds we have found support previous hypotheses by Gao and Ye [16], *i.e.*, that UV-absorbing compounds are responsible for protection of photosynthesis in *N. flagelliforme*. Those authors claimed the need for further investigations on this aspect. Here we expand their findings, obtained with field experiments, using a different experimental approach. We performed laboratory tests with a simulated solar radiation specifically deprived of UV-wavebands with cut-off filters. Using PSII activity as a most sensitive physiological parameter we give a direct proof of the effectiveness of UV-protective compounds. The experimental procedures we propose can lead to a standardisation of methods to compare UV-protection in organisms which differ in their complement of UV-sunscreens. In this concern, we show the same experiments performed in a laboratory-grown *Nostoc commune* lacking UV-absorbing compounds.

## 2. Results and Discussion

### 2.1. Complement of UV-absorbing compounds in *Nostoc flagelliforme*

**Figure 1:**
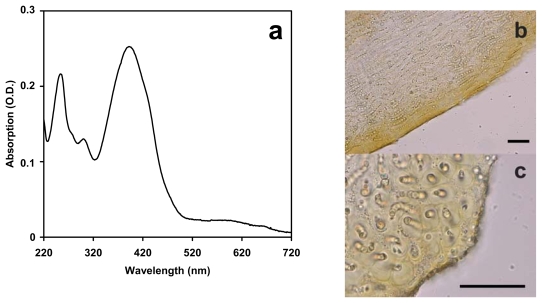
(a) Absorption spectrum of an extract from *N. flagelliforme* in 80% tetrahydrofuran. (b–c) Micrographs of sections (10 μm thick) of *N. flagelliforme* showing the peripheral localization of yellow-brown scytonemin. Scale bar: 50 μm. Longitudinal section (b); cross section (c).

Extraction of *N. flagelliforme* with 80% tetrahydrofuran yielded an extract with the main characteristics of scytonemin absorption (Figure 1a). This pigment absorbs most strongly in the UV-A (315–400 nm), but there is also significant absorbance in the visible region, especially in the violet and blue range as well as in the UV-B (280–320 nm) and UV-C (190–280 nm) [9]. Scytonemin is a water-insoluble pigment localized in the sheath covering the cyanobacteria. Light microscopy of sections of *N. flagelliforme* clearly showed the distribution of yellow-brown scytonemin in the peripheral region of the filaments (Figure 1b–c).

**Figure 2:**
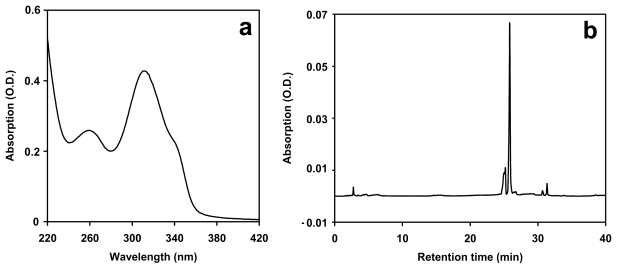
(a) Absorption spectrum of an extract from *N. flagelliforme* in 20% methanol. (b) Chromatogram of UV-absorbing putative OS-MAAs from *N. flagelliforme*. Lichrospher RP-18 (5 μ, 250*4 mm I.D), 1 mL min^−1^, 0–15 min: 100% A (0.02% acetic acid in water). 15–30 min: linear increase from 0% B (acetonitrile) to 20% B.

Extraction with 20% methanol yielded an extract with an absorption peak at 311 nm and a shoulder at around 335 nm (Figure 2a). These absorption characteristics are very similar to those of OS-MAAs [8]. *N. flagelliforme* was also extracted in pure water to release loosely bound OS-MAAs or with an aqueous solution of *N*-acetylcysteine (NAC) to destabilize the glycan sheath [23] and thus potentially release a more tightly bound fraction of OS-MAAs. These extracts showed similar UV-absorption (λ_max_ 310 nm in water and 311 nm in NAC solution) but the absorption at the maximum was only about 20% of the methanol extracts for water extracts and about 61% for NAC extracts.

The release of UV-absorbing compounds by extraction with water at room temperature was also found in field-grown *N. commune* samples, but not in laboratory-grown strains in liquid culture [7]. The enhanced release of putative OS-MAAs upon treatment with the mucolytic agent NAC is in agreement with the localization of these compounds in the extracellular glycan sheath. HPLC separation of putative OS-MAAs from *N. flagelliforme* showed no correspondence to MAA standards of shinorine, porphyra-334, palythine or palythinol (Figure 2b). The total amount of UV-absorbing putative OS-MAAs was calculated from the 20% methanol extracts as 32.1 mg g DW^−1^ (sd = 1.9 mg g DW^−1^), using the extinction coefficient of 17 cm^2^ mg^−1^ at 312 nm [8].

In the *N. commune* strain supposedly deprived of UV-sunscreens, the same extraction procedures did not yield any absorption peak of UV-screening compounds (not shown). The fact that *N. commune* was not exposed to UV-radiation prior to the experiments, together with the absence of a conspicuous glycan sheath, explains the absence of MAAs in this strain. In contrast, the field-grown samples of *N. flagelliforme* had been exposed to natural solar radiation in their habitat before collection. Interestingly, the amount of OS-MAAs found in *N. flagelliforme* by far exceeds the values that were reported in *N. commune* collected during spring in Southern Germany (7 mg g DW^−1^) [8]. The more than fourfold concentration of putative OS-MAAs in *N. flagelliforme* as compared to field-collected *N. commune* from Southern Germany can be attributed to genetic differences as well as to the different climate at the collection sites. The two sites, *i.e.*, Sunite Zuoqi and Southern Germany, differ strongly in the regime of precipitation and solar irradiation. The climate in Southern Germany is more humid, with about 750 to 950 mm yearly precipitation, as compared to less than 300 mm at the semi-arid collection site of *N. flagelliforme* in Sunite Zuoqi, Inner Mongolia (data from the GPCC Homepage: http://gpcc.dwd.de).

Scytonemin as well as the putative OS-MAAs considerably absorb radiation throughout the UV-A and UV-B range. The protective effect of UV-absorbing compounds on diverse vital parameters, including photosynthetic carbon fixation [24], has been demonstrated in a wide range of organisms [24–26]. In *N. flagelliforme* the combination of compounds having complementary absorption maxima at 370 nm, corresponding to *in vivo* absorption of scytonemin [9], and 312/335 nm, corresponding to MAAs [27], is very likely to induce the photosynthetic insensitivity to UV-radiation recently described by Gao and Ye [16]. In addition to the mutual shading of cells in the multiserate filaments of *N. flagelliforme*, the predominant localization of scytonemin in the outer layer is likely to increase the protection of this organism from harmful UV-radiation [25].

### 2.2. Fluorescence quenching analysis

In *N. flagelliforme*, the photosynthetic capacity was fully recovered after at least 16 h rehydration in BG-11 medium, with a kinetics (not shown) comparable to that reported in literature [19–21]. The rehydrated trichomes were used for studies on the response of photosynthesis to UV radiation. The above mentioned strain of *N. commune* was also studied. The two strains were preliminarily analysed in order to ascertain if their acclimation characteristics to PAR were comparable.

Quenching of chlorophyll fluorescence provides a useful means to characterise the response of cyanobacteria to PAR. Besides PSII photoinhibition, cyanobacteria have two mechanisms that contribute to non-photochemical quenching of chlorophyll fluorescence: state transitions, with consequent energy dissipation through PSI, and phycobilisome-related energy dissipation [28]. In fact, the assumption of Campbell and coworkers that non-photochemical quenching in cyanobacteria is dominated by state transitions [29] has been rectified by recent experiments showing carotenoid-triggered energy dissipation in phycobilisomes [30]. Nevertheless, that assumption remains a useful approximation for the interpretation of the changes in the *NPQ* coefficient under increasing irradiance (see Table 1 for definition of fluorescence parameters and coefficients).

**Figure 3:**
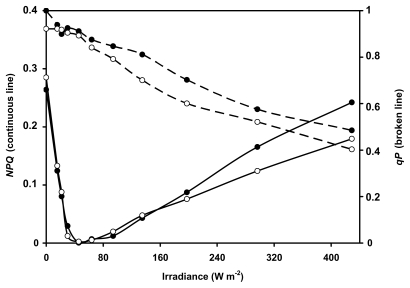
Non-photochemical quenching (*NPQ*, continuous lines) and photochemical quenching (*qP*, broken lines) of chlorophyll fluorescence in *N. flagelliforme* (closed circles) and *N. commune* (open circles) under increasing light intensity.

The two *Nostoc* species developed marked *NPQ* in darkness due to the respiratory reduction of the electron chain [31] (Figure 3). In this condition, energy is driven to photosystem I (PSI) and the fluorescence excited by the saturation pulse (*F_M dark_*_)_ is relatively low. As light is applied, PSI oxidises the chain, energy is driven mainly to PSII, maximum fluorescence (*F_M_*′) increases and *NPQ* decreases. When incident light exceeds the acclimation light, energy begins again to be driven to PSI and *NPQ* increases. The two species showed similar irradiance-*NPQ* curves with a minimum value at approximately 45 W m^−2^, corresponding to the optimal light intensity for acclimated growth of the organisms (Figure 3) [29].

The irradiance-response curve of *qP* was also similar in the two species and showed a decline of the coefficient under increasing irradiance (Figure 3). The *qP* value reflects the proportion of open PSII in the thylakoid membrane. *N. flagelliforme* and *N. commune* are similar in their ability to maintain the PSII open also under high irradiance, which is common in cyanobacteria, and mainly due to the high capacity of these organisms to remove electrons from PSII. This PSII buffering ability has at least two components, one due to the oxidising activity of PSI, the other to the cytochrome oxidase [32].

Collectively, the results of the quenching analyses showed that the two species did not significantly differ in their response to PAR at the level of the photosynthetic membranes. Therefore, it seems that the high irradiance environment of *N. flagelliforme* has not selected a special acclimation ability of PSII to high PAR in this organism with respect to the laboratory-grown *N. commune*. This surprising finding might be explained by the fact that *N. flagelliforme* in its natural habitat starts photosynthesis in the early morning when it is partially rehydrated by dew [17] and becomes progressively dehydrated during the morning hours, in parallel with increasing irradiance.

### 2.3. Protection of PSII activity in *Nostoc flagelliforme* in the UV-radiation range

The decay in PSII quantum yield, a useful measure of PSII activity [33], was analysed in *N. flagelliforme* during a 2-h exposure to a Hönle-lamp simulating solar radiation. Incident radiation included high intensity PAR and UV-radiation. Starting from full radiation, cut-off filters were used to deprive the incident radiation of UV-wavebands. The condition of only PAR was obtained with a 395-nm cut-off filter.

**Figure 4:**
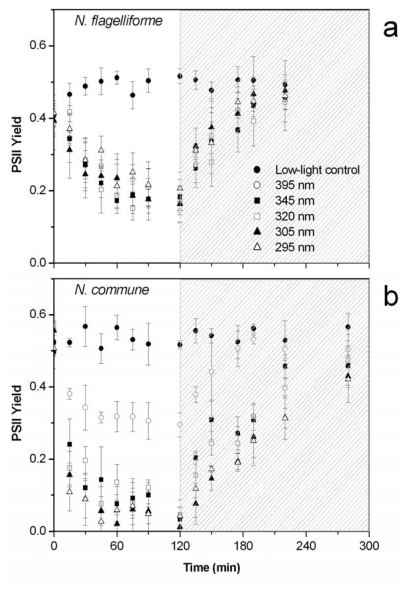
PS II quantum yield during exposure of *N. flagelliforme* (a) and *N. commune* (b) to a solar lamp using different cut-off filters (indicated in the legend). The shaded areas indicate recovery times after transfer to dim light. Each point represents the mean ±SD (*n* = 4–5).

In *N. flagelliforme* all treatments lowered the PSII yield as compared to the low-light control; significant differences between the 395 nm cut-off filter and the shorter wavelength cut-off filters were absent (Figure 4a).

The same experiment was performed with the laboratory-grown non-UV-adapted cyanobacterium, *N. commune*. Similar to *N. flagelliforme*, in *N. commune* the decay in PSII quantum yield observed with the 395 nm cut-off filter was only due to the exposure to high PAR (Figure 4b). However, when UV components were also present, the PSII quantum yield was strongly affected and reached nearly the zero value after 2 h exposure. The control and 395 nm cut-off filter treatments were significantly different from the treatments receiving lower wavelength radiation at all exposure times up to 120 min (*P* < 0.01). No wavelength-dependent behavior in response to different UV components was evident (only after 15, 45 and 60 min significant differences were found between individual cut-off filters in the UV-range, but no consistent pattern was obvious).

**Figure 5:**
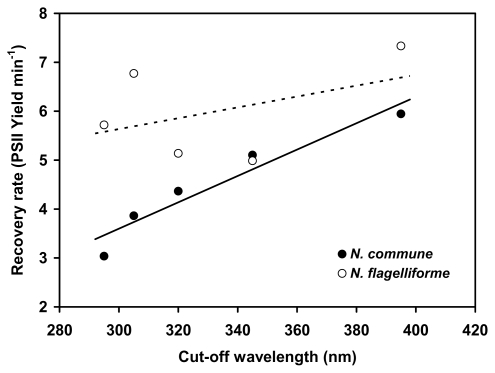
Linear regression of the initial recovery rate of PSII yield after transfer to dim light with the cut-off wavelength of the filters used during simulated solar irradiation.

In the UV-sensitive strain, the difference in the extent of PSII photoinhibition induced by PAR or PAR + UV-radiation, independently of the UV cut-off filter used, shows that the UV-A radiation alone (345 nm cut-off filter) is sufficient to induce a marked inhibition of PSII photochemical activity. Consequently, at the end of the irradiation period, the value of PSII quantum yield in itself did not allow us to discriminate a specific UV-B-induced effect. Conversely, additional damage due to the exposure to UV-B radiation is expected to cause slowed recovery rates of PSII quantum yield, because the UV-B-damaged PSII requires more complex steps for reactivation than the PAR-damaged PSII [14,34]. Since repair of UV-induced photodamage of PSII occurs under low visible light [34,35], after the simulated solar irradiation the *Nostoc* samples were exposed to low light intensity to trigger the recovery of PSII yield. In the UV-sensitive *N. commune* the recovery rate highly correlated with the wavelength of the cut-off filters (*r^2^* = 0.9236, *P* < 0.01), very likely reflecting the complex metabolism of PSII, in which the turnover of D1 protein of the photochemical reaction center is differently affected by PAR and UV radiation depending on wavelength [14]. Interestingly, in *N. flagelliforme* there was only a weak correlation between the initial recovery rate of PSII quantum yield and cut-off wavelength (*r^2^* = 0.1858, *P* = 0.47) (Figure 5). Therefore, it appears that in this organism the photoinhibition of PSII is essentially due to high light, with no additional effect of UV radiation. The extent of PSII photoinhibition induced by high PAR in *N. flagelliforme* was comparable to that of the UV-sensitive *N. commune*, confirming the results obtained with the fluorescence quenching analysis, *i.e.*, *N. flagelliforme* does not have special acclimation properties of PSII to high PAR.

**Figure 6:**
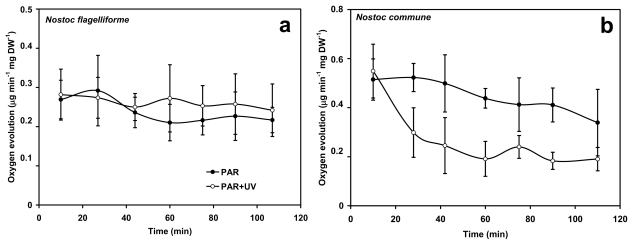
Oxygen evolution of *N. flagelliforme* (a) and *N. commune* (b) during exposure to a solar lamp either deprived of the UV-components of its spectrum (PAR) or not (PAR + UV). Each point represents mean ±SD (*n* = 4–5).

To draw conclusions on the effectiveness of UV-protection of photosynthesis in *N. flagelliforme*, we also analysed the PSII activity in terms of oxygen evolution. Two conditions were considered, *i.e.*, PAR + UV (cut-off at 295 nm) and exclusively PAR (cut-off at 395 nm). In *N. flagelliforme* no significant differences between samples exposed to PAR + UV and only PAR were found (Figure 6a). As expected, in *N. commune* oxygen evolution was significantly lower in UV-treated samples as compared to only high-PAR treatment throughout the experiment, except during the first 10 min and at the end of the experiments (*P* < 0.05) (Figure 6b). Interestingly, on a DW basis, the oxygen evolving capacity under only PAR of *N. flagelliforme* was half the value obtained with *N. commune*. This can be mainly attributed to the extracellular materials of the glycan sheath, which contribute a substantial part of the DW in *N. flagelliforme*.

## 3. Conclusions

Scytonemin and MAAs are compounds that are considered to have appeared early in the history of life. After their appearance in marine organisms, they represented an adaptive benefit for the colonization of terrestrial environments exposed to higher levels of UV-radiation. The usefulness of these marine drugs in a genuinely terrestrial organism as *N. flagelliforme* is an example of the significance of marine bioactive compounds for life on Earth. The use of simulated solar irradiation specifically deprived of certain wavebands in combination with PAM fluorescence and oxygen evolution measurements has proven to be a suitable approach to the characterization of the impact of PAR and UV-radiation on photosynthesis in strains of cyanobacteria that are differently adapted to radiation stress. The response of *N. flagelliforme* to UV-radiation under controlled conditions shows that its UV-insensitivity covers the whole UV-A and UV-B range and is almost certainly due to the complementary UV-absorption of MAAs and scytonemin [27]. This response is even more outstanding if compared with that of a *Nostoc* strain lacking detectable amounts of such substances.

MAAs are especially abundant and concentrated in the glycan sheath in the form of oligosaccharide-linked compounds. The special properties of OS-MAAs including their ability to interact with complex macromolecular organic matrices have not yet been explored for their use in practical applications such as cosmetic UV-sunscreens. The results presented in this research justify the increasing scientific interest on the photoprotectants synthesized by cyanobacteria and encourage further research efforts to clarify, among others, the details of their synthesis and its regulation and their further potential for use as UV-protective products.

## 4. Experimental Section

### 4.1. Experimental organisms

*Nostoc flagelliforme* (Berkeley and Curtis) Bornet and Flahault was a gift from Prof. Kunshan Gao, Xiamen University, derived in dried form from a field collection in Sunite Zuoqi (Inner Mongolia/China) in May 2004. The *N. commune* strain used for parallel analyses had originally been isolated from a rice paddy field near Varanasi (India) and was routinely grown in BG-11 medium [36] in Erlenmeyer flasks placed near the laboratory window, where the cultures were exposed to diffuse, window-glass filtered daylight.

### 4.2. Extraction and analysis of UV-absorbing compounds

Water soluble UV-absorbing compounds were extracted from air-dry specimens of *N. flagelliforme* either with MilliQ-water, with 20% methanol in water, or with a 30 mM solution of N-acetylcysteine (NAC) in water at room temperature. Every sample (*n* = 3–4) was extracted subsequently four times in equal volumes of solvent for 1 h. The extracts were scanned from 200 to 450 nm with a spectrophotometer (DU-70, Beckman, USA). The extracts were directly analyzed by HPLC using a reversed phase column (Lichrospher RP-18, 250 * 4 mm I.D.) and a gradient using 100% of solvent A (0.2% acetic acid in water) from 0 to 15 min and a linear increase from 0% solvent B (acetonitrile) to 20% B from 15 to 30 min at a flow rate of 1 mL min^−1^. For spectrophotometric estimation of the amount of UV-absorbing putative OS-MAAs, a linear baseline tangential to the absorption spectrum at 280 nm and 375 nm was subtracted. The extinction coefficient of 17 cm^2^ mg^−1^ at 312 nm [8] was used for estimating the concentration of putative OS-MAAs. Scytonemin was extracted with 80% tetrahydrofuran in water overnight at 4 °C.

### 4.3. Microscopy

Dry filaments of *N. flagelliforme* were embedded in polyethylene glycol (PEG 4000/PEG 600 3:1) and sectioned on a sledge microtome (Leitz, Wetzlar, Germany) to sections of 10 μm thickness. The sections were immersed in BG-11 medium to rehydrate the samples and to remove the PEG. After at least 3 h of rehydration the sections were observed in bright field illumination using a Keyence BZ-8000K microscope (Keyence, Osaka, Japan) equipped with a Nikon Plan Apo 20X/0.75 objective.

### 4.4. Modulated chlorophyll fluorescence: Preparation of samples and equipment

For Pulse Amplitude Modulated (PAM) chlorophyll fluorescence analyses, small mats of *N. flagelliforme* were rehydrated in small, uncovered Petri dishes with BG-11 medium at 25 °C and with 10 W m^−2^ irradiance for at least 16 h and not more than 48 h before being used for analyses. During the measurements, the rehydrated filaments of *N. flagelliforme*, as well as flakes of *N. commune*, were maintained with a small aliquot (300–500 μL) of medium. A portable fluorometer PAM 2000 (Walz, Effeltrich, Germany) was used for measurements [33]. The fluorometer uses a complex fiber optics probe for guiding the measuring light and the saturating pulse to the sample; the PAM probe was placed perpendicularly at a distance of about 1 mm from the sample to yield a sufficient signal from the cells. For quenching analyses, the actinic light was directed to the sample using the same probe.

### 4.5. Irradiance-response curves of chlorophyll fluorescence quenching

Irradiance-response curves of chlorophyll fluorescence were obtained following the procedure proposed by Campbell *et al*. [29]. The samples were dark-adapted for 5 minutes and then *F_0_* was determined by illuminating the sample with the low-intensity measuring light (600 Hz, 665 nm). Subsequently, *F_M dark_* was determined with a 0.8-s pulse of saturating white light (measuring light set to 20 kHz). After 30 s darkness, the actinic light was turned on with the lowest intensity, until the steady state level *F_t_* was reached (typically, 2 min). Subsequently, the actinic light was turned off and the sample was irradiated with weak far red light for 5 s, thus allowing PSI to extract electrons from the transport chain. *F_0_*′ was evaluated as the lowest of 5 determinations. The actinic light was then resumed and the *F_t_* was re-established. With a new pulse *F_M_*′ was determined. The actinic light intensity was increased and the measuring procedure was repeated sequentially to generate a light-response curve.

The fluorescence parameters were used for the calculation of quenching coefficients. The non-photochemical quenching of chlorophyll fluorescence was determined through the *NPQ* coefficient, according to the Stern-Volmer formulation [37]:

(1) $$NPQ=\frac{F_{M}-{F_{M}}^{\prime }}{{F_{M}}^{\prime }}$$

This equation was preferred to other formulations because it is independent of *F_0_* and thus does not suffer distortion by underlying phycobiliprotein fluorescence [29]. Since absolute levels of fluorescence were not critical to this work, the highest *F_M_*′ was assumed as an approximation of the actual *F_M_*. Indeed, the prediction of the acclimated light intensity simply depends on the pattern of *NPQ* in response to light intensity. Acclimated light intensity corresponds to an “integrated light information” over time based on which the organism regulates the synthesis of abundant proteins of the photosynthetic system [29]. The light intensity at which minimum *NPQ* is achieved coincides approximately with the acclimated light intensity [29]. The photochemical quenching of variable chlorophyll fluorescence was determined as [38]:

(2) $$qP=\frac{{F_{M}}^{\prime }-F_{t}}{{F_{M}}^{\prime }-{F_{0}}^{\prime }}$$

The *qP* coefficient quantifies the actual fraction of PSII reaction centers that are in the open state. It reflects the balance between excitation of PSII centers, which close them, and removal of electrons from PSII by the electron transport chain, which reopens the centers.

### 4.6. Effect of UV irradiation on the PSII quantum yield

The samples were exposed to a Hönle lamp (irradiance: PAR 102 W m^−2^; UV-A 24 W m^−2^; UV-B 0.4 W m^−2^) and cut off filters at 295, 305, 320, 345, and 395 nm were used to deprive the simulated solar radiation of different components of the spectrum. The outer surface of the dishes was painted black, to avoid reflectance phenomena during the exposure to the lamp. Samples (*n* = 4–5) were exposed for 120 min and the PSII quantum yield was measured at 15 min intervals. Control samples exposed to low fluorescent light (10 W m^−2^) were measured in parallel. After the exposure period, the samples were allowed to recover under low fluorescent light for 100 min. The same experiment was performed using flakes of *N. commune*; in this case, the recovery under low light was monitored for 160 min. The effective PSII quantum yield was calculated after measuring *F_t_* and *F_M_*′ [38,39]:

(3) $$Y=\frac{{F_{M}}^{\prime }-F_{t}}{{F_{M}}^{\prime }}$$

The initial recovery rate of PSII quantum yield was estimated by fitting the recovery kinetics with a 2^nd^ order polynomial function using the Microsoft Excel 2000 software:

(4) $$Y=at^{2}+Rt+Y_{0}$$

where *Y* is PSII yield and *t* the recovery time. *R* coefficient represents the initial recovery rate of PSII yield (*Y* min^−1^). The rate is decreasing as a function of time (according to the *a* coefficient in the equation) until the *Y* plateau value is reached.

### 4.7. Oxygen evolution measurements

The photosynthetic oxygen evolution was measured with the Visual OXYM instrument (Real Time Computers, Möhrendorf, Germany), which is designed to analyse oxygen evolution in phytoplankton, macroalgae or aquatic plants. The instrument uses standard Clark electrodes and sends data, after amplification and A/D conversion, to a host computer. OXYM is controlled by the *μVOXYM* software supplied with the instrument. The instrument used for the measurements in this research was a five channel-type, which allows one to measure oxygen evolution in up to five samples at the same time. Electrodes were calibrated using nitrogen-bubbled water (zero oxygen) and air-bubbled water (oxygen saturated water). Rehydrated *N. flagelliforme* filaments (ca. 9 mg DW) were cut into 3–4 mm-long fragments and put into the cuvettes, which were then filled with BG-11 medium. For measurement of *N. commune*, flakes of the organism, suspended in the BG-11 medium as uniformly as possible, were put into the cuvettes. After the measurements the dry weight of the samples was determined (4–7 mg). The cuvettes of the OXYM are UV-transparent, as well as one side of the water jacket where the cuvettes are located. A refrigerator system based on a water circuit (in this case connected to a thermostat) allows the maintenance of a stable temperature in the water jacket (19 °C). The instrument is also endowed with a light sensor which monitors the irradiance reaching the samples for each measured point. The illumination source was a Hönle lamp (irradiance at the level of the samples: PAR 172 W m^−2^; UV-A 46 W m^−2^; UV-B 0.95 W m^−2^). Radiation from the lamp was filtered or not with a 395 nm cut off filtering sheet in order to screen some samples from the UV component of the spectrum. Before starting the irradiation, samples were dark-adapted for at least 10 min. Irradiation lasted for 120 min and throughout the experiment the samples were stirred by means of small magnetic stirrers included in each cuvette. The sampling rate was set at 5 s per point.

### 4.8. Statistics

Mean oxygen evolution rates for 10 min exposure intervals were compared by Student’s *t*-test between UV-treated samples and PAR-only samples, using the *t*-test function of *Microsoft Excel*. In the PAM-experiments, where more than two different radiation treatments were applied, simple factorial ANOVA was applied separately for each exposure time, followed by Tukey HSD test, using the ANOVA program developed by Brown [40].

## Figures and Tables

**Table 1:** Definition of fluorescence parameters and coefficients.

Parameter	Definition
*F_0_*	Minimum fluorescence in the dark-adapted state
*F_0_*′	Minimum fluorescence in the light-adapted state
*F_M dark_*	Maximum fluorescence in the dark-adapted state
*F_M_*′	Maximum fluorescence in the light-adapted state
*F_M_*	Maximum fluorescence estimated as the highest *F_M_*′ in irradiance-response curves
*Ft*	Steady-state fluorescence
*NPQ*	Non-photochemical chlorophyll fluorescence quenching
*qP*	Photochemical quenching of variable chlorophyll fluorescence
*Y*	Actual quantum yield of photochemical energy conversion in PSII
